# Post Caesarean Ogilvie’s Syndrome

**DOI:** 10.5334/jbr-btr.905

**Published:** 2016-08-30

**Authors:** Aurore Margot, Hamoir Xavier

**Affiliations:** 1 ISPPC, BE; 2 Hopital Notre Dame Tournai, BE

**Keywords:** Ogilvie, intestinal obstruction, caesarean

A 35-year-old woman underwent a caesarean at 39 weeks and 6 days of her pregnancy. The operation was done without complications. Over the next days, the patient complained about abdominal bloating and nausea. Clinical examination noted a decrease in peristalsis. The biological analysis showed an inflammatory syndrome with a C-Reactive Protein (CRP) level of 143 mg/L and hyperneutrophilia up to 13930/µl. Abdominal contrast-enhanced Computed Tomography (CT) was performed and revealed distension (with air-fluid levels) of the stomach, the small bowel and both ascending and transverse colon (Figure 1). The descending and sigmoid colons showed no sign of distension. No transitional mechanical obstacle could be found. Furthermore, the presence of a pneumoperitoneum was to be seen in the light of the history of her recent caesarean section. The diameter of the caecum was 11 cm. Post caesarean Ogilvie’s syndrome was suspected and consecutively colonoscopy was carried out three days after delivery. It showed dilation of the transverse and right colonic lumen for which exsufflation was accomplished. There was an ulcerated and necrotic layer in the lower caecum towards the hepatic flexure of the colon. A close biological and clinical monitoring was recommended. The next day, the patient still complained about abdominal bloating. A plain abdominal X-ray showed an significant pneumoperitoneum and hydroaeric levels in the intestines (Figure 2). The patient underwent right hemicolectomy; anatomopathology revealed ceacal wall necrosis.

**Figure 1:**
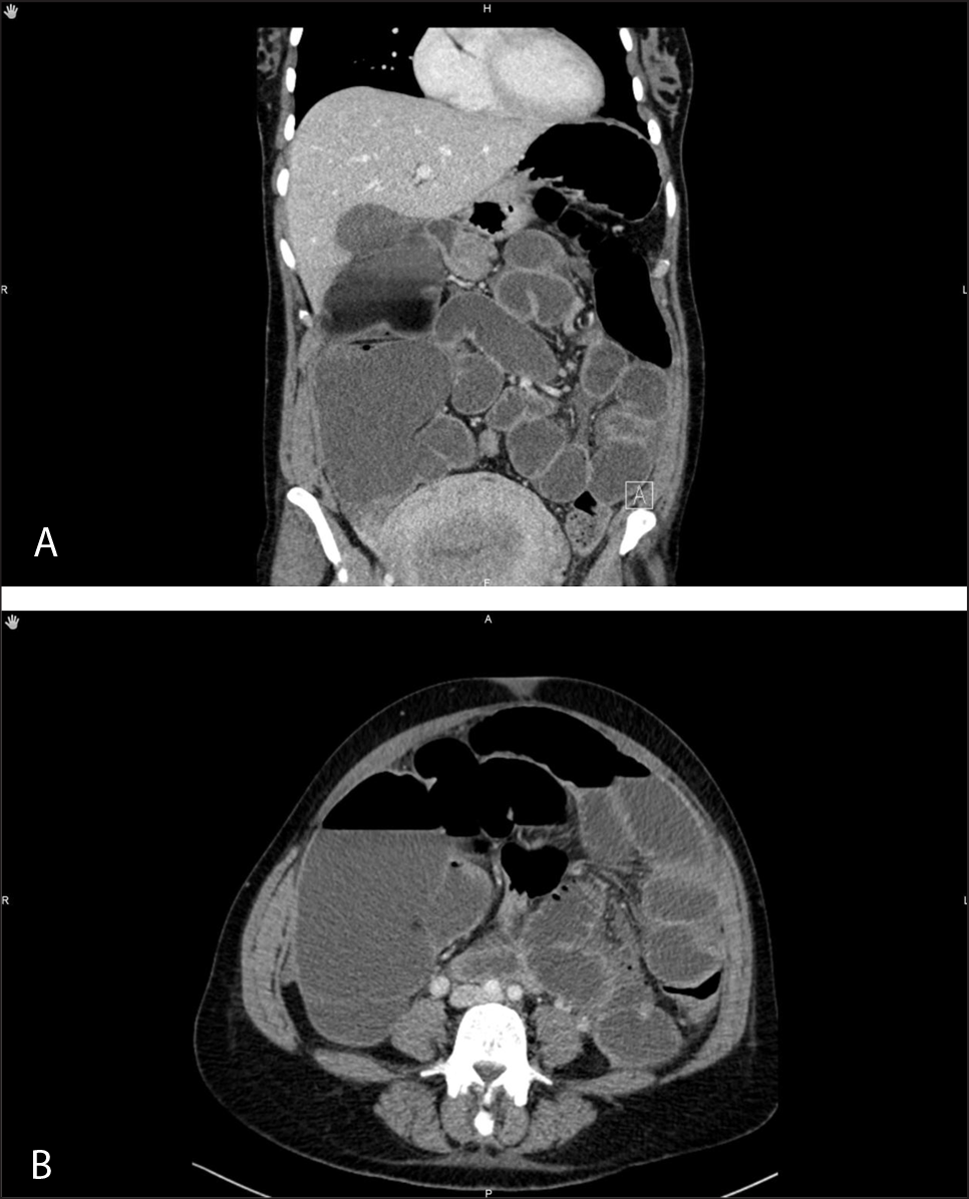
Abdominal contrast-enhanced CT revealing distension (with air-fluid levels) of the stomach, the small bowel and both ascending and transverse colon.

**Figure 2:**
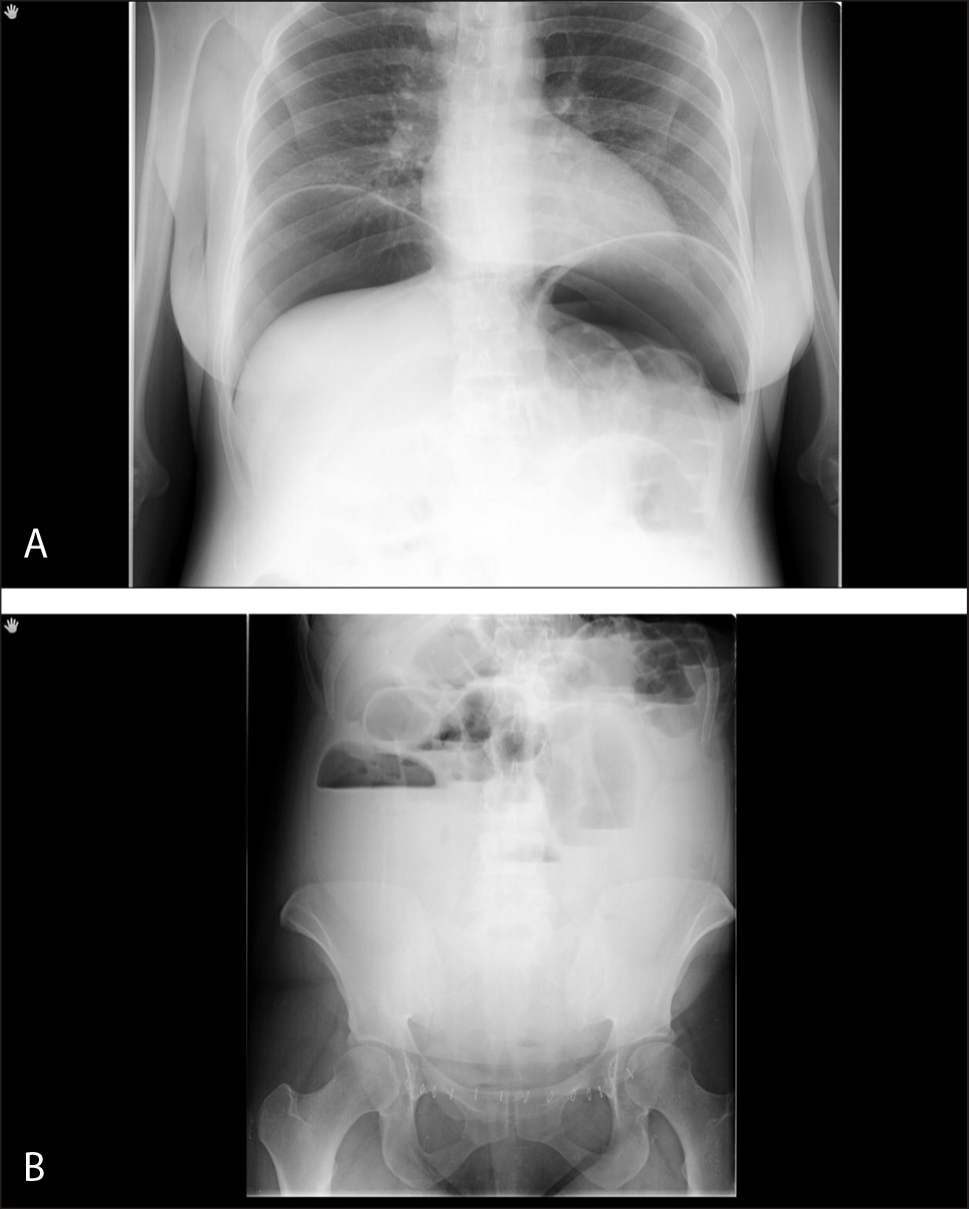
Plain abdominal X-ray showing an important pneumoperitoneum and hydroaeric levels in the intestines.

## Comment

Ogilvie’s syndrome is also called acute colonic pseudo obstruction (ACPO) and is a clinical condition with symptoms, signs and radiographic appearance of acute large bowel obstruction, mostly right and transverse, without a mechanical cause. ACPO may be linked with a defect of the parasympathetic innervation of the left colon causing a loss of its peristalsis, which would lead to upstream dilation and hyperpressure within the right colon. In young women, caesarean section is the most common surgical procedure associated with the onset of this syndrome that occurs post-operatively at an average of 4–5 days. The clinical features of ACPO include lower incomplete intestinal obstruction and mostly abdominal distension [1]. Plain abdominal radiograph is sufficient to infer the diagnosis by showing colon distension, sometimes associated with air-fluid levels. The presence of colon haustra, rectal gas and the scarcity of hydroaeric levels may differentiate ACPO from mechanical obstruction. CT may help ruling out mechanical obstruction as in the present case. Therapy is based on several treatment modalities. It is assumed that when the caecum diameter is less than 9 cm, patients can undergo medical treatment first, via parasympathetic analogues. Colonoscopic exsufflation often helps to obtain the decompression of the colon, but carries significant risks for perforation. In case of perforation or intestinal distress, surgery is obviously the standard treatment.
